# Internet Cognitive Behavioral Therapy With or Without Face-to-Face Psychotherapy: A 12-Weeks Clinical Trial of Patients With Depression

**DOI:** 10.3389/fdgth.2020.00004

**Published:** 2020-06-19

**Authors:** Katrin Rauen, Stefan Vetter, Amanda Eisele, Ewelina Biskup, Aba Delsignore, Michael Rufer, Steffi Weidt

**Affiliations:** ^1^Department of Geriatric Psychiatry, Psychiatric Hospital Zurich, University of Zurich, Zurich, Switzerland; ^2^Department of Psychiatry, Psychotherapy, and Psychosomatics, Psychiatric Hospital Zurich, University of Zurich, Zurich, Switzerland; ^3^Department of Neurology, University Hospital Zurich, Zurich, Switzerland; ^4^Department of Advanced Biomedical Sciences, Frederico II University of Naples, Naples, Italy; ^5^College of Fundamental Medicine, Shanghai University of Medicine and Health Sciences, Shanghai, China; ^6^Faculty of Medicine, University of Zurich, Zurich, Switzerland

**Keywords:** depression, ICBT, face-to-face psychotherapy, QoL, BDI-II, WHOQOL-BREF, trial

## Abstract

Depressive disorders are a curable, global health problem. However, most patients remain untreated, and more and more patients use internet-based interventions, but it is unclear whether it is beneficial for ongoing face-to-face psychotherapy. Thus, we compared the outcome of internet cognitive behavioral therapy (ICBT) with (ICBT+) or without (ICBT) additional face-to-face outpatient psychotherapy in adult patients with moderate to severe depressive disorder. For this longitudinal interventional clinical trial (NCT02112266), 168 of 252 online recruited adults with depressive symptoms received ICBT+ (*n* = 96) or ICBT (*n* = 72). Demographics (sex, age, age at first depressive episode, years of education, duration of depressive symptoms) were assessed and compared between groups. All patients underwent ICBT for 12 weeks. Quality of life (QoL) and severity of depressive symptoms were assessed within each group at three time points [baseline (T0), postinterventional after ICBT at 12 weeks (T1), and for follow-up at 6 months (T2)] using the World Health Organization Quality of Life Questionnaire (WHOQOL-BREF) global score to assess QoL as primary and the Beck Depression Inventory (BDI-II) to assess self-rated depressive symptoms as secondary outcome variables, respectively. Differences were assessed between groups using *t* test and over time using repeated-measures analysis of variance. Data of intention-to-treat analysis are given as mean ± SD. Group differences were assumed at *p* < 0.05. Partial η^2^ is given as effect size. Demographic data, QoL, and depressive symptoms did not differ between groups (ICBT+/ICBT) at baseline (T0). Patients of both groups suffered from moderate to severe depressive disorders and gained improved QoL scores (WHOQOL-BREF-global: *p* < 0.001, η^2^ = 0.16), as well as experienced decreased depressive symptoms (BDI-II: *p* < 0.001, η^2^ = 0.2) after 12 weeks of ICBT compared to baseline. Patients without additional face-to-face outpatient psychotherapy lost QoL—albeit not significant—and had increased depressive symptoms (BDI: *p* = 0.02, η^2^ = 0.04) at 6 months' follow-up. Thus, ICBT is suitable for psychiatric treatment, although additional face-to-face outpatient psychotherapy helps stabilizing long-term outcome.

## Introduction

Depressive disorders are a major, though curable, global health problem that remarkably hampers patient's quality of life (QoL). The World Health Organization (WHO) has ranked major depression worldwide the third cause of burden, and it is estimated to be the primary cause by 2030 (1, 2). The lifetime risk is 15–18%, and women are twice as often affected as men (3, 4). However, prior to puberty, affective disorders are equally distributed between sexes, but notably, this sex- and gender-related difference in the prevalence of depression occurs during adolescence and remains stable over the life span, indicating besides genetic factors a role of sex hormones and/or gender-related educational issues for the pathogenesis (5, 6). Depression is similarly prevalent in high- vs. middle- and low-income countries indicating that causes go far beyond modern lifestyle and poverty (2, 7, 8). Nevertheless, a remarkable number of patients remain undiagnosed (9–11), and almost half remain untreated (12, 13). Reasons for not seeking professional help are unawareness with failure of recognizing depressive symptoms, the limited capacities of therapists, and the persisting tenacious stigma of mental disorders (10, 12–14). This treatment gap is especially relevant in the young, thereby increasing the risk of progression in terms of recurrence and aggravation of episodes in adulthood, and endures despite the large body of effective non-pharmacological and pharmacological treatment (10, 15–17).

Psychotherapy effectively tackle mild to moderate depressive disorders, whereas moderate to severe depression needs a combined approach of pharmacotherapy and psychotherapy (18). To date, cognitive behavioral therapy (CBT) is the most evidence-based psychotherapy for depression (13, 19), and patients often prefer non-pharmacological rather than pharmacological or combined approaches. Recently, online therapies gained more attention to close the current treatment gap for depressive disorders (13, 20, 21). Internet-based cognitive behavioral interventions, such as the internet cognitive behavioral therapy (ICBT), improve mild to moderate depressive symptoms and have been shown to be beneficial when compared to usual care either by the general practitioner or a psychotherapist without grading of the evidence [Grading of Recommendations Assessment, Development and Evaluation (GRADE) not conducted] and in comparison to waiting-list patients with moderate evidence (GRADE moderate) (22). However, there is also the contrary opinion and evidence that ICBT might be inferior regarding individual and face-to-face outpatient CBT (21). Nevertheless, the evidence of ICBT improving severe depressive symptoms is rare (23), and to our knowledge, ICBT has not yet been compared to ICBT plus face-to-face psychotherapy in moderate to severe depression (22). Thus, evidence regarding QoL outcome and relief of symptoms in moderate to severe depressive patients has not yet been fully explored.

Quality of life is a suitable and increasingly applied subjective outcome measure to assess patient's well-being over time. The WHO defines QoL as “an individual's perception of their position in life in the context of the culture and value systems in which they live and in relation to their goals, expectations, standards, and concerns. It is a broad-ranging concept with complex interactions with a person's physical health, psychological state, personal beliefs, social relationships, and their relationship to salient features of their environment,” (24) thereby emphasizing the relevance of internal and environmental factors and its interplay for good QoL.

Currently, more and more internet-based interventions are available and are used by patients with or without ongoing face-to-face psychotherapy. Nevertheless, it is unclear whether these internet-based interventions are favorable or even unfavorable due to different therapy approaches at the same time. Therefore, we investigated the outcome of ICBT with or without additional face-to-face outpatient psychotherapy in adult patients with a moderate to severe depressive disorder.

## Patients and Methods

### Study Design and Patients

This longitudinal interventional preregistered clinical trial (NCT02112266) was conducted online. A multipronged approach included several recruitment strategies, namely, announcements on depressive disorder websites, postings in online self-help forums, and notices in chat rooms for depressive symptoms. Patients were enrolled online from April 2014 until March 2016. After the presurvey, potentially eligible subjects received a participation code. All online questionnaires were programmed using QuestBack Unipark (25). After final study inclusion, patients were assigned to the ICBT group receiving exclusively the ICBT online therapy or to the ICBT+ group receiving ICBT plus additional face-to-face outpatient psychotherapy. All patients underwent ICBT for 12 weeks without study site visits, thus from T0 until T1. Therefore, time points of assessments were at baseline (T0), after 12 weeks of the ICBT treatment (T1), and at 6 months after the start of the ICBT treatment for one follow-up (T2). Demographics (sex, age, age at first depressive episode, years of education, duration of depressive symptoms) were assessed and compared between groups at baseline (T0). Changes of QoL as primary outcome were measured by the World Health Organization Quality of Life Questionnaire (WHOQOL-BREF) global score. Depressive symptoms were quantified with the BDI-II as secondary outcome. Primary and secondary outcomes were assessed over time at three time points, that is, at baseline (T0), after the intervention of ICBT at 12 weeks (T1), and at 6 months' follow-up (T2). The study was approved by the local ethics committee of the Canton of Zurich in Switzerland (KEK-ZH-Nr. 2013-0542). It was conducted in full accordance with the Declaration of Helsinki, with all subjects providing their electronic informed consent prior to participation and the EQUATOR/CONSORT standards.

### Inclusion and Exclusion Criteria

Patients aged 18–65 years of both sexes (female/ male) were eligible for study inclusion if depressive symptoms were at least moderate to severe with a BDI-II equal to or >20 and <40, and symptoms persisted at least for 2 weeks. German language was required. Exclusion criteria were very severe depression according to a BDI-II of beyond 40, suicidal ideation, alcohol or drug dependency, history of psychotic symptoms, history of bipolar disorder, current inpatient care, or semiresidential treatment.

### Intervention

Both groups received the ICBT over 12 weeks, developed by makora AG (www.makora.ch). Subjects were allocated to this internet intervention, including eight different modules, which were released every week. All modules were based on psychoeducation and exercises for the following topics: (1) symptom recognition, (2) identification of reasons for depressive symptoms, (3) increasing positive activation, (4) thought observation, (5) thought identification, (6) error in reasoning resolving, (7) social activity improving, and (8) relapse preventing. There was no personal study support other than technical. However, subjects received automatic e-mails when (i) they did not finish an ICBT module, (ii) did not log in into the program for more than 7 days, and (iii) a new module was released to work on. After the 12 weeks of ICBT, thus between the end of the ICBT (T1) and the follow-up at 6 months (T2), subjects could use the ICBT program without any restrictions. During this period, they received no more automatic e-mails but were occasionally reminded that they were taking part in a study. There were neither recommendations nor restrictions during the 3-months period from the end of the structured and guided ICBT (T1) to the follow-up, thus at 6 months after the start of the trial (T2). Therefore, all participants were able to continue or discontinue ICBT in accordance to their individual motivation and preferences, and patients of the ICBT+ group could follow their individual needs regarding the face-to-face psychotherapy.

### Demographics and Outcome Measures

Sociodemographic and clinical data were obtained through structured questions about sex (female/male), age, age at first depressive episode, years of education, and duration of depressive symptoms and were compared between groups at baseline (T0).

The WHOQOL-BREF in its German version was used to assess QoL for primary outcome over time within and between groups (26). The WHOQOL-BREF is a validated patient-reported outcome instrument assessing the patient's global health and well-being within the recall period of 2 weeks and has been developed to provide a validated short form covering 26 items and all facets of the WHOQOL-100—one of the most applied QoL assessments. Despite its significant reduction in questions compared to the WHOQOL-100, the WHOQOL-BREF is a sound, cross-culturally valid instrument with good to excellent psychometric properties with the advantage of quick completion (26, 27). The WHOQOL-BREF generates a QoL profile of four domains, namely, physical health, psychological, social relationships, environment, and two further items, that is, the individual's overall perception and the global health with a score from 0 to 100 representing worst and best QoL, respectively.

The BDI-II was used for secondary outcome measures assessing depressive symptoms over time within and between groups. The BDI-II is a self-assessment and can be completed within 5 to 10 min (28, 29). The questionnaire consists of 21 items, each rated on a 4-point scale ranging from 0 to 3, representing no to severe symptoms within the past 2 weeks. Responses were summed, yielding a score between 0 and 63, with higher scores indicating severest depressive symptoms. In detail, a score from 0 to 8 represents no clinical signs of depression; a score of 9 to 13, minimal; a score of 14 to 19, mild; a score of 20–28, moderate; and a score of 29–63, severe depression. The BDI-II is reliable with a Cronbach α of 0.93 for depressed patients and has a good internal and external test validity with *r* = 0.72–0.89 and *r* = 0.68–0.70, respectively. Instruments' responsiveness is along reliability and validity one out of the three measurement properties to determine the quality of a health-related patient-reported outcome and represents the capability to detect the patient status' changes over time (30, 31)

### Statistics

Descriptive statistics were used to characterize subjects regarding demographics and therapy characteristics of the ICBT and the ICBT+ group. Differences (demographics, WHOQOL-BREF global score, BDI-II) at baseline were assessed per group using *t* test for independent samples and over time using repeated-measures analysis of variance. Intention-to-treat analysis with first observations carried forward was conducted for the dependent variables WHOQOL-BREF global score and the BDI-II. Group (ICBT vs. ICBT+) served as the between-subject variable, with time (pre, post, follow-up) as the within-subject factor. Data are given as mean ± SD. Outliers were removed if data exceeded more than 2 SD. Group differences were assumed at *p* < 0.05. To determine effect sizes, a partial η^2^ was calculated, indicating a small (η^2^ ≥ 0.01), medium (η^2^ ≥ 0.06), or large effect (η^2^ ≥ 0.14) (32). Statistical calculations were performed using SPSS (version 25.0; IBM, Armonk, New York, USA). Graphs were illustrated using Prism8 (GraphPad Software, San Diego, CA, USA) (33).

## Results

**Figure 1:**
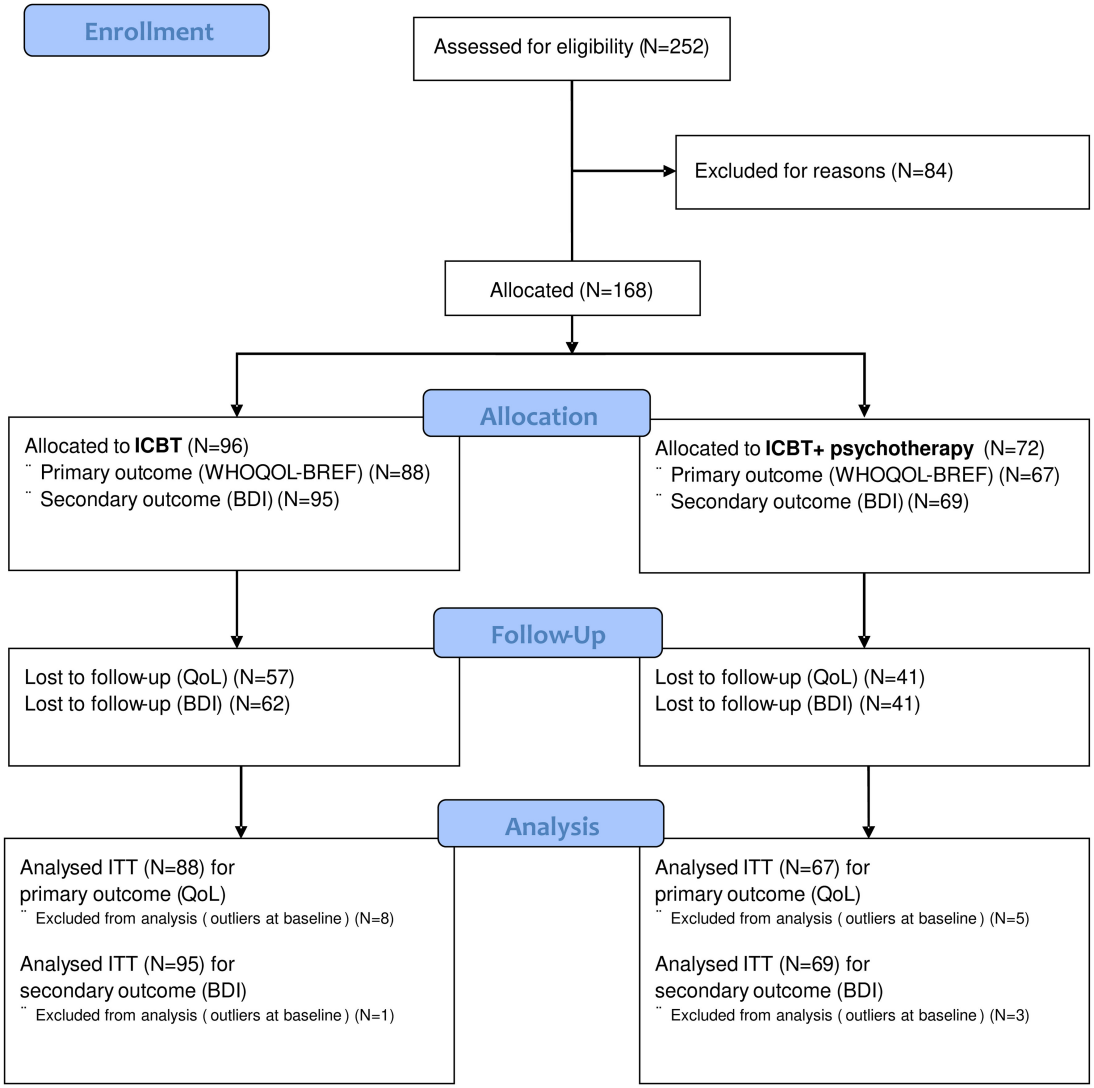
Flowchart of enrollment and analysis for ICBT.

Two hundred fifty-two online recruited adults with depressive symptoms were assessed for eligibility (Figure 1). Eighty-four patients were excluded according to the exclusion criteria: current suicidal ideation (*n* = 26), inpatient treatment (*n* = 2), alcohol or drug dependency (*n* = 7), psychotic symptoms (*n* = 2), bipolar disorder (*n* = 4), not given e-mail address (*n* = 27), and not willing to participate (*n* = 16). Thus, 168 of 252 (67%) received participant codes to enter the online therapy. Of those, 96 (57%) had no outpatient psychotherapy and were allocated to the ICBT group, whereas 72 had outpatient treatment for their depressive disorder and were assigned to the ICBT+ group. After removal of eight and five outliers, respectively, 88 patients of the ICBT and 67 patients of the ICBT+ group were analyzed for their primary outcome (WHOQOL-BREF global score). For secondary outcome (BDI-II), 95 patients of the ICBT, and 69 patients of the ICBT+ group were analyzed after removing one and three outliers, respectively.

### Demographics and Therapy Characteristics

Demographics, namely sex (female/ male), age, age at first depressive episode, years of education, and duration of depressive symptoms, were assessed at baseline and did not differ between groups, that is, the ICBT (*n* = 96) and the ICBT+ (*n* = 72) group (Table 1).

**Table 1:** Demographics at baseline.

**Demographics**	**ICBT group (*N* = 96)**	**ICBT + psychotherapy (*N* = 72)**	***p*-value^#^**
Sex (females/males) (%)	76%/24%	79%/21%	0.71
Age in years (mean ± SD)	35.4 ± 11.5	37.5 ± 11.9	0.26
Age at first depressive episode in years (mean ± SD)	21.0 ± 9.6	21.7 ± 14.3	0.73
Years of education (mean ± SD)	14.6 ± 5.4	15.9 ± 5.0	0.11
Depressive symptoms in weeks (mean ± SD)	68.2 ± 159.7	73.4 ± 151.0	0.83

# *t-test after Shapiro-Wilk-Test for normality*.

The ICBT and ICBT+ groups did not statistically differ regarding (i) the total time spent for ICBT, (ii) the total counts of logins, (iii) the counts of logins between T0 and T1, and (iv) the counts of logins between T1 and T2. In detail, total time spent for ICBT was on average in the ICBT group 4.5 ± 6.3 h (mean ± SD) and in the ICBT+ group 5.7 ± 9.3 h (*p* = 0.36). The total count of logins was on average 11.5 ± 12.6 within the ICBT group compared to 12.7 ± 14.8 (*p* = 0.62) within the ICBT+ group. Between T0 and T1, subjects of the ICBT group had 10.7 ± 11.4 compared to 11.3 ±12.3 logins of the ICBT+ subjects (*p* = 0.79). During T1 and T2, the count of logins was 0.78 ± 1.8 within the ICBT and 1.1 ± 2.9 within the ICBT+ group (*p* = 0.46), representing the low tendency to continue the ICBT, that is, only 19 and 15 subjects, respectively. Frequencies of face-to-face psychotherapy within the ICBT+ group were as follows: 43.7% of subjects received face-to-face psychotherapy at least once per week, 42.3% one to two times per month, and 14.1% less frequent.

### Quality-of-Life Outcome

**Figure 2:**
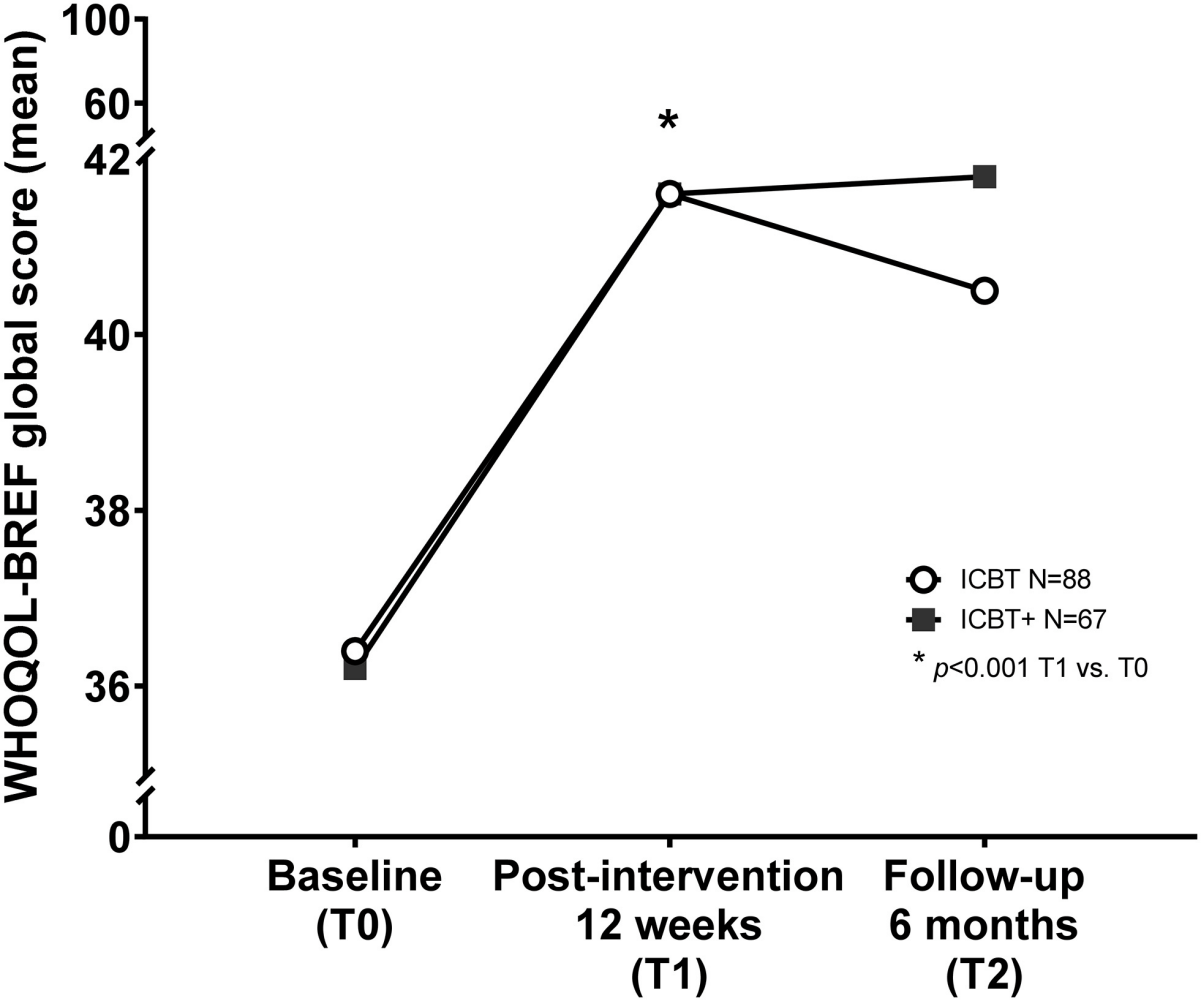
Internet cognitive behavioral therapy and quality of life outcome. Internet cognitive behavioral therapy (ICBT) is suitable to improve quality of life (QOL) measured by the WHOQOL-BREF global score within 12 weeks of treatment (*p* < 0.001, η^2^ = 0.16). Patients who received additional face-to-face outpatient psychotherapy (ICBT+) were able to stabilize their gained QoL up to 6 months after the start of the intervention, whereas patients without additional face-to-face psychotherapy slightly lost QoL, although not significant. *indicates the significant longitudinal improvement of QoL between baseline (T0) and after 12 weeks of treatment (T1), and thus between T0 and T1 within both groups.

The WHOQOL-BREF global scores did not differ between groups (ICBT: *n* = 88/ ICBT+: *n* = 72) at baseline (36.4 ± 13.9/36.2 ± 11.9; *p* = 0.94) (Figure 2 and Table 2). Intention-to-treat analysis comparing WHOQOL-BREF global scores at T0 and T1 revealed significant improved QoL within groups (*p* < 0.001) with a large effect size indicated by a partial η^2^ = 0.16, but without group differences (*p* = 0.87; η^2^ < 0.01). Subjects of the ICBT group reported reduced QoL at 6 months (T2) compared to the end of the ICBT at 12 weeks (T1), albeit this observation was not significant within groups (*p* = 0.62, η^2^ < 0.01) or between groups (*p* = 0.49, η^2^ < 0.01).

**Table 2:** Influence of ICBT on quality of life outcome.

**Primary outcome**	**Baseline (T0)**		**Post-intervention 12 weeks (T1)**		**Follow-up 6 months (T2)**		**Group difference**	**Differences over time within groups**	
	**ICBT (*N* = 88)**	**ICBT+ (*N* = 67)**	**ICBT (*N* = 88)**	**ICBT+ (*N* = 67)**	**ICBT (*N* = 88)**	**ICBT+ (*N* = 67)**	**ICBT vs. ICBT+**	**T0 vs. T1**	**T1 vs. T2**
WHOQOL-BREF global (mean ± SD)	36.4 ± 13.9	36.2 ± 11.9	41.6 ± 18.0	41.6 ± 15.0	40.5 ± 18.1	41.8 ± 16.4	*p* = 0.87 η^2^ < 0.01	*p* < 0.001 η^2^ = 0.16	*p* = 0.62 η^2^ < 0.01

*ITT analysis (N = 155) General linear models (repeated measures)*.

*η^2^: effect size partial eta squared (≥ 0.01 small effect, ≥ 0.06 medium effect, ≥0.14 large effect)*.

### Depressive Symptoms

**Figure 3:**
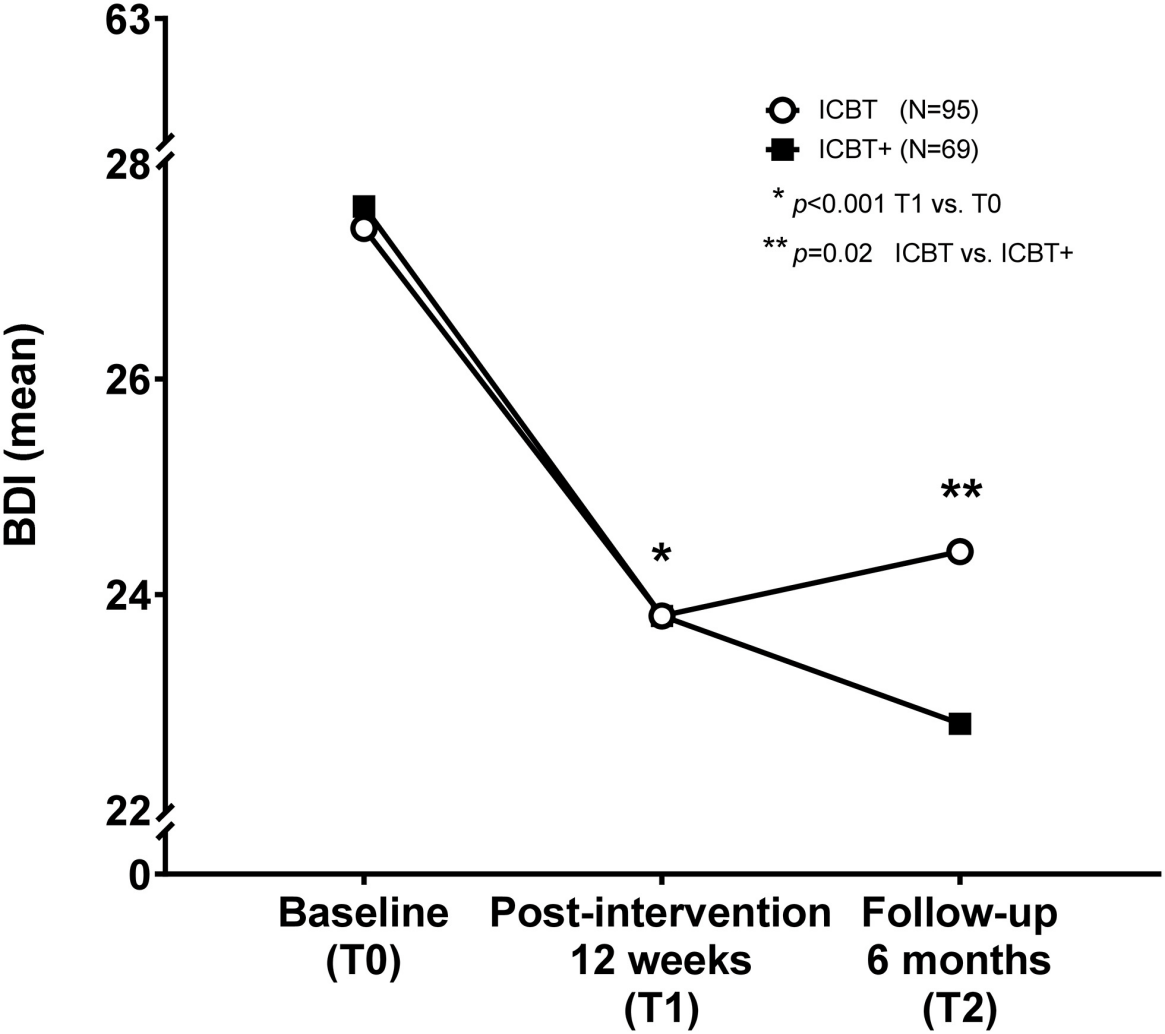
Internet cognitive behavioral therapy and depressive symptoms. Internet cognitive behavioral therapy (ICBT) is suitable to improve depressive symptoms measured by the Beck Depression Inventory in its revised version (BDI-II) within 12 weeks of treatment (*p* < 0.001, η^2^ = 0.2). Patients who received additional face-to-face outpatient psychotherapy (ICBT+) further improved and reported fewer depressive symptoms, whereas those without additional psychotherapy deteriorate up to 6 months after the intervention (*p* = 0.02, η^2^ = 0.04). Thus, ICBT with additional face-to-face outpatient psychotherapy seems to be beneficial for patients to reduce depressive symptoms in the long run. *indicates the significant longitudinal decrease of depressive symptoms between baseline (T0) and after 12 weeks of treatement (T1), and thus between T0 and T1 within both groups. **indicates the significant difference between ICBT and ICBT+ groups with respect to time from T1 to T2, thus the end of the guided ICBT treatment for both groups and the 6 months follow up with significant less depressive symptoms in patients having received ICBT plus face-to-face psychotherapy.

The BDI-II scores did not differ between groups (ICBT: *n* = 95/ ICBT+: *n* = 69) at baseline (27.4 ± 7.7/27.6 ± 7.1; *p* = 0.97) (Figure 3 and Table 3). Intention-to-treat analysis comparing BDI-II scores at T0 and T1 underlines reduced depressive symptoms in both groups with *p* < 0.001 indicated by a large effect size with a partial η^2^ = 0.2. Subjects of the ICBT group showed slight deterioration of depressive symptoms, with higher BDI-II scores compared to those subjects of the ICBT+ group at 6 months (T2) and compared to the end of the ICBT at 12 weeks (T1) (*p* = 0.02, η^2^ = 0.04). Thus, BDI-II scores differed within groups over time from T0 to T1, and between groups over time from T1 to T2, indicating a beneficial effect of additional face-to-face outpatient psychotherapy.

**Table 3:** Impact of ICBT on depressive symptoms.

**Secondary outcome**	**Baseline (T0)**		**Post-intervention 12 weeks (T1)**		**Follow-up 6 months (T2)**		**Group difference**	**Differences over time within groups**	
	**ICBT (*N* = 95)**	**ICBT+ (*N* = 69)**	**ICBT (*N* = 95)**	**ICBT+ (*N* = 69)**	**ICBT (*N* = 95)**	**ICBT+ (*N* = 69)**	**ICBT vs. ICBT+**	**T0 vs. T1**	**T1 vs. T2**
**BDI (mean** **±** **SD)**	27.4 ± 7.7	27.6 ± 7.1	23.8 ± 10.4	24.0 ± 9.6	24.4 ± 10.6	22.8 ± 10.6	*p* = 0.77 η^2^ < 0.01	*p* < 0.001 η^2^ = 0.2	*p* = 0.35 η^2^ < 0.01

*ITT analysis (N = 164) General linear models (repeated measures)*.

*η^2^: effect size partial eta squared (≥ 0.01 small effect, ≥ 0.06 medium effect, ≥ 0.14 large effect)*.

## Discussion

This longitudinal interventional clinical trial included adult patients aged 18–65 years of both sexes with moderate to severe depressive disorders investigating QoL outcome measured by the WHOQOL-BREF global score for primary and changes of self-assessed depressive symptoms measured by the BDI-II for secondary outcome. Demographics and outcome measures, that is, QoL and BDI scores, did not differ at baseline between the two investigated groups, that is, the ICBT and ICBT+ groups, the latter having received additional face-to-face outpatient psychotherapy, whereas the ICBT group received solely the online treatment of eight modules on psychoeducation and exercises. Patients of both groups, namely, ICBT and ICBT+, reported improved QoL and had reduced self-assessed depressive symptoms after 12 weeks (T1) of online treatment with or without additional face-to-face outpatient psychotherapy, and this result did not differ between the two groups. At follow-up (T2), namely, 6 months after the trial's start, there was a trend that patients receiving additional face-to-face outpatient psychotherapy (ICBT+) were able to stabilize their QoL, whereas lack of face-to-face outpatient psychotherapy (ICBT) might result in reduced QoL over time. In terms of self-assessed depressive symptoms at 6 months' follow-up, patients receiving exclusively online treatment slightly deteriorated, whereas patients with additional face-to-face outpatient psychotherapy further improved over time and reported significantly fewer depressive symptoms compared to the ICBT patients. These results suggest that additional face-to-face outpatient psychotherapy may have helped stabilizing outcome over time. Internet cognitive behavioral therapy can help improve QoL and depressive symptoms especially for those patients having limited access to psychotherapy and/or being afraid of psychiatry-related stigma, thereby being supportive to overcome lack of treatment capacities or stigma of psychiatric consultations.

### Demographics

The participating patients most probably suffered from recurrent depressive disorders with onset during their adolescence and were within their third life decade with a female-to-male ratio of 4:1. From the literature, it is well-known that first episodes of depressive disorders occur from midadolescence to mid-40s, with almost half of patients experiencing first depression before the age of 20 years with peaks in the second and third decades of life (2); thus, our study cohort is well in line with the literature regarding age at study participation and age at first depressive episode. Regarding the female-to-male ratio, our study cohort had three times more women than men; thus, women are overrepresented in our study compared to the prevalence of depression mostly given in the literature (2–4, 6, 34). However, socioeconomic factors play a role for depression, and significant cross-national variations with higher percentage of women, thus comparable to our cohort, have been reported (35). As the prevalence of depression changes between females and males during adolescence and remains stable over the life span, the biological sex due to hormonal changes and differences of brain structures as well as gender-related factors are relevant (6, 35). It is especially noteworthy that the association of higher education with better mental health is significantly more relevant for women than for men in Europe (35). Why most probably an overrepresented number of women participated in our study remains unclear, but it can be speculated that females rather than males recognize depressive symptoms and might be more self-aware of symptoms, and thus seeking help more frequently. Future research should focus on how to better reach males and younger participants suffering from depression (e.g., with specific advertisement for online treatment for these subgroups). Based on our results, specific suggestions for additional face-to-face outpatient psychotherapy might be offered to participants during the ICBT training.

### Quality of Life and Depression

Quality of life was connotatively hampered by moderate to severe depression in our patients and was significantly improved in both groups by ICBT. However, norm values of QoL measured by the WHOQOL-BREF have been described beyond a score of 70 (36), albeit norm data for the global score are missing. However, the international validation of the WHOQOL-BREF included 11,830 subjects and revealed mean values of 3–4 on the 5-point scale for the general QoL and general health with moderate correlations with the four domains, that is, physical, psychological, social, and environment of the WHOQOL-BREF (27, 37). Furthermore, other commonly used health-related QoL scores, e.g., the SF-36, have well-reported norm values for, e.g., the German population with mean scores of approximately 50 on a scale ranging from 0 to 100, the latter representing best QoL (38). Thus, most probably QoL of our patient cohort was still hampered after ICBT with or without additional face-to-face outpatient psychotherapy. Most likely, this impeded QoL is influenced by the long average duration of depressive symptoms; that is, patients of our cohort have cumulatively suffered more than 4 years from depressive symptoms. A second major reason might be the early onset of depression during adolescence; thus, our participating patients may live in a disadvantageous environment or even have experienced an adverse life event; hence, epigenetic factors might play a role and might limit treatment success (2, 39, 40).

Depressive symptoms abated from an initially moderate to severe to a lower moderate level over the 12 weeks of ICBT with or without additional face-to-face outpatient psychotherapy. The follow-up period of 6 months revealed a further decrease of depressive symptoms in patients having additional face-to-face outpatient psychotherapy, whereas those patients without significantly deteriorated—albeit patients of both groups remained moderate depressive over the period of 6 months. Therefore, additional face-to-face psychotherapy is strongly recommended for improved long-term results. However, our results emphasize the need for more intense and better therapies including physical activities, environmental approaches, and pharmacotherapy for chronic depressive patients amenable to online therapies as overall results are quite disappointing.

### Cognitive Behavioral Therapy and Depression

Cognitive behavioral therapy is the most widely available and best evident psychotherapy to treat patients with depression (2, 41). Yet, approximately half of patients do not have access to treatment because of unawareness of the disease, lacking capacities of psychotherapists, or the persisting stigma of mental health disorders (10, 12–14). Thus, a large body of internet-based therapies is available. Previous studies recommend ICBT to improve mild to moderate depression, but treatment success merely persisted over a short period of time; namely, from 6 to 24 months after the ICBT, no group differences were obvious when compared to an active control condition (42). This previous reported unsatisfactory long-term effect of ICBT is well in line with our current results. Therefore, we suggest that future studies need to evaluate patients' behavioral and environmental long-term changes and the effort and time invested in the online treatment after structured and guided ICBT is applied. Why the effect of ICBT remained relatively small in our cohort cannot fully be explained. Nevertheless, the usually rapid effect of ICBT can be hampered by personality disorders (21, 42). As our patient cohort experienced first depressive episodes during adolescence with a high probability of chronic depression, it is possible that at least part of the cohort suffers from a personality disorder with negative impact on outcome. One major problem in treatment of mental disorders is the patient's individual engagement as up to one-third of patients discontinue treatment, and of those who entered continuation phase, 40% break off (13, 20, 43).

### Limitations

There are several limitations of our study that warrant discussion. First, data on previous and current therapies including pharmacotherapy were not available or incomplete (43), which limits the interpretability and the discussion of our results. Second, details on the additional face-to-face outpatient psychotherapy including the total amount of treatment hours, the therapeutic focus, and the patient's individual capabilities to change maladaptive behaviors or detrimental cognitive beliefs were incomplete or not assessed. These factors need future considerations not only because patients with depression having received twice-weekly compared to once-weekly psychotherapy are suggested to have an improved outcome (44), but also to better anticipate the face-to-face psychotherapeutic effect beyond its frequency. Third, data on the total time spent on face-to-face or ICBT treatment during the treatment and follow-up period are incomplete, a weakness that is known from the literature and needs to be assessed in future studies (45). Fourth, loss to follow-up was quite high, although representing the common and well-known problem of depressive patients' motivation and engagement for treatment continuum (43, 44). Fifth, we did not analyze a control group without any treatment or included a waiting group; therefore, it cannot fully be excluded that time influenced at least part of the patient's improvement (41). However, there is evidence that ICBT is superior over control groups and is not only an alternative to watchful waiting (46, 47). Furthermore, application of ICBT is superior to waiting-list groups with a moderate evidence level (GRADE moderate) (22), and guided ICBT can be as effective as face-to-face CBT (48). Sixth, we did not assess environmental factors that might interfere with treatment effects and need to be included in future studies.

### Future Perspectives

Digital health is rapidly nascent, and a variety of future perspectives need considerations. First, the most important direction that needs to be tackled is the use of (i) chatbots using artificial intelligence and machine learning rather than decision trees, (ii) embodied conversational avatars, or even (iii) hackathons that have the potential to increase patients' therapy adherence, responsiveness, beneficial behavioral patterns, and social interactions to overcome depression and to pave the way to personalized medicine in mental health (49–57). Second, objective outcome measurements are important to emphasize the beneficial effect of online therapy trials using neuroimaging, e.g., diffusion tensor imaging and functional magnetic resonance imaging, which can help to elucidate microstructural changes in the white matter tracts of the interhemispheric and frontal-subcortical neural circuits that are impaired in depression (58), or to depict the striatal hypoactivation of the reward neuronal network within unipolar depression when anticipating and consuming rewards (59), or epigenetic and modifiable factors, e.g., the dense DNA methylation status of the glucocorticoid receptor gene (NR3C1), which is associated with early life stress and disposes to major depression disorder (60). Third, environmental factors such as previous and current negative life conditions, marital status, manifestation, and duration of dysfunctional beliefs might play a role for therapy adherence in ICBT (61); thus, we advocate to include besides emerging digital strategies and objective measures more environmental factors in future studies.

## Conclusion

During ICBT, QoL and depressive symptoms improved in patients suffering from moderate to severe depression. At 6 months' follow-up, patients having received ICBT and additional face-to-face outpatient psychotherapy were able to stabilize their improved QoL in contrast to diminished QoL in patients with sole ICBT and reported significantly reduced depressive symptoms at the lower border of moderate severity, compared to slight deterioration of the ICBT patients. These results suggest additional face-to-face outpatient psychotherapy may help stabilize outcome over time, and structured and guided ICBT supports patients having limited access to face-to-face psychotherapy and/or being afraid of psychiatry-related stigma. For long-time effects, the continuous treatment of structured and guided ICBT and/or additional face-to-face psychotherapy is recommended.

## Data Availability Statement

All relevant data for this study are included in the article. The raw data supporting the conclusions of this article will be made available by the authors, without undue reservation, to any qualified researcher.

## Ethics Statement

The study was approved by the local ethics committee of the Canton of Zurich in Switzerland (KEK-ZH-Nr. 2013-0542). The participants provided their electronic informed consent prior to participation in this study.

## Author Contributions

SW conceptualized, initiated, designed, and supervised the entire study. KR conceptualized the data analysis, supervised the statistical data analysis, gave important, intellectual content, interpreted the data, wrote the abstract for the pre-submission, wrote the manuscript, and illustrated graphs and tables. SV revised the manuscript and gave important, intellectual content. AE performed the statistical analysis, revised the final manuscript. EB revised the manuscript and gave important, intellectual content in terms of sex- and gender related aspects. AD helped to initiate and design the study, revised the final manuscript. MR helped to initiate and design the study, revised the final manuscript and gave important intellectual content. All authors approved the final version of the manuscript and agreed to be accountable for the content of the work.

## Conflict of Interest

The authors declare that the research was conducted in the absence of any commercial or financial relationships that could be construed as a potential conflict of interest.

## Abbreviations

BDI-II: Beck Depression Inventory, 2nd version
CBT: Cognitive Behavioral Therapy
GRADE: Grading of Recommendations Assessment, Development and Evaluation
ICBT: Internet Cognitive Behavioral Therapy
ICBT+: Internet Cognitive Behavioral Therapy plus additional face-to-face outpatient psychotherapy
η^2^: Partial eta squared (effect size)
QoL: Quality of Life
SD: Standard Deviation
WHO: World Health Organization
WHOQOL-BREF: World Health Organization Quality of Life Questionnaire.
